# Changes in Tight Junction Protein Expression Levels but Not Distribution in Commercial White and Brown Laying Hens Supplemented with *Chondrus crispus* or *Ascophyllum nodosum* Seaweed

**DOI:** 10.3390/ani14050777

**Published:** 2024-03-01

**Authors:** Leslie A. MacLaren, Jingyi Wang, Shima Borzouie, Bruce M. Rathgeber

**Affiliations:** Department of Animal Science & Aquaculture, Dalhousie University, Truro, NS B2N 5E3, Canada; jingyiwang@dal.ca (J.W.); borzouie@dal.ca (S.B.); brathgeber@dal.ca (B.M.R.)

**Keywords:** chicken, prebiotic, seaweed, tight junction, jejunum, occluding, ZO-1, genetic strain

## Abstract

**Simple Summary:**

The intestinal epithelium is the layer of cells that line the digestive tract, and it acts both as a selective filter to facilitate the absorption of nutrients and a protective barrier to toxins and infection. The functional integrity of this constantly renewing cell layer depends on so-called tight junction proteins, a class that includes two regulatory proteins called occludin and zonula occludens-1 (ZO-1). We studied the effect of two different prebiotic seaweed supplements for 41 weeks on occludin and ZO-1 expression in two genetic lines of laying hen. Differences between genetic lines in the level of occludin in a highly absorptive section of the small intestine called the jejunum were found. There also were differences due to seaweed supplementation in the ZO-1 protein, and red seaweed supplementation appeared to have more impact in the brown laying hen strain than in the white strain. Overall, our results are consistent with previous studies showing genetic differences in diet response.

**Abstract:**

It is proposed that prebiotic diet supplements improve intestinal function, in part by improving the barrier function of the intestinal epithelium with an associated increase in the expression of tight junction proteins, including occludin and zonula occludens-1 (ZO-1). We examined the expression of these proteins in two strains of laying hens (Lohman LSL-lite (White) and Lohman Brown-lite (Brown)) who were supplemented or not with 3% *Chondrus crispus* or 0.5% *Ascophyllum nodosum* seaweeds from 31 to 72 weeks of age. Occludin was localized to the lateral surfaces and across the intestinal epithelium in all animals. Reactivity for ZO-1 was concentrated at the apicolateral epithelial cell membrane border. Mood’s median test indicated that White hens may express more occludin in villus epithelium (median intensity 3.5 vs. 2.5 in Brown hens, *p* = 0.06) but less ZO-1 in the deep cryptal epithelium (median intensity 1.5 vs. 2.5 in Brown hens, *p* = 0.06). Western blotting also showed higher levels of occludin in White than Brown hens (*p* < 0.05). A decrease in ZO-1 Western blot expression was associated with *Chondrus crispus* supplementation in comparison to controls (*p* < 0.05), but not with *Ascophyllum nodosum* supplementation (*p* > 0.05). In conclusion, genetic strain and dietary seaweed supplements affect tight junction regulatory protein expression levels but do not impact the anatomical distribution, as seen in cryosections.

## 1. Introduction

In both mammals and birds, the intestinal epithelial barrier is integral to the regulation of absorption and secretion, as well as resistance to pathogens and toxins [1,2]. The latter may damage the intestinal epithelial cells directly or affect the tight junctions that normally resist transport between adjacent enterocytes [3]. The degree of paracellular permeability is regulated so that the tight junctions may form pores that increase ‘leakiness’ [3]. Originally discovered in chickens as localizing to the tight junctions, occludin (OCC) is a transmembrane protein that associates with zonula occludens-1 (ZO-1), a peripheral membrane protein, both of which are involved in the regulation of tight junctions and intestinal epithelial turnover [4,5,6]. More specifically, OCC mediates proliferation and signaling for apoptosis of enterocytes, and upregulation is associated with increased barrier function [4,6]. ZO-1 regulates apical membrane structure and epithelial cell proliferation under stress [6]. 

Pre- and probiotic supplements are of interest due to their observed beneficial effects on digestive health in humans and domestic animals, including poultry. The microbiome of the large intestine is a major target of such supplements, but the small intestine is also affected, where it is difficult to differentiate direct vs. microbiome-mediated effects [1,3]. Providing prebiotic carbohydrate to obese or diabetic mice decreased the intestinal epithelial permeability and expression of inflammatory markers and increased the tight junction integrity, as well as the expression of OCC and ZO-1 mRNA [4]. The jejunum is the major site for absorption of macronutrients in the mammalian and avian small intestine, and its morphology has been shown to respond positively to pre- and probiotics in laying hens [7,8]. Supplementation of eight-day old piglets with galacto-oligosaccharides (GOS) increased jejunal expression of OCC and ZO-1, as well as indicators of intestinal enzyme and transport function [9]. Providing GOS to broiler chickens prevented heat shock induction of heat shock proteins and inflammatory interleukins in jejunum and prevented increases in ZO-1 associated with heat shock; the effects were different than those observed in the ileum [10]. Similarly, supplementation of broiler chickens with a multistrain probiotic was shown to mitigate the effects of Salmonella challenge on the expression of OCC in the jejunum [11].

Most prebiotic seaweed supplement studies to date have been short-term feeding trials in broilers and other domestic species [12]. We have been interested in the potential health benefits of seaweed supplements in laying hens over their productive life. The red seaweed *Chondrus crispus* improved performance and increased intestinal villus height in the ileum of commercial layers in short-term trials [13,14]. In a 41-week trial, we showed that red or brown (*Ascophyllum nodusum*) seaweed supplementation affected plasma protein and enzyme levels as well as performance, and their response to heat stress depended upon the genetic strain [14,15]. Supplemental marine-derived polysaccharides improved production, egg quality and indicators of jejunal function when fed to 62-week-old laying hens for 6 weeks [8]. A 14-week study of laying hens’ response to a green microalgae, *Desmodesmus* spp., showed changes in jejunal brush border activity as well as other digestibility effects but no effect on performance [16]. Little is known about genetic differences in intestinal permeability in poultry, but it has been shown that there are breed differences in intestinal villus structure and cell profile, as well as OCC expression [17,18]. 

Given the observations in broilers and other species showing effects of pre- and probiotic supplements on stress response and indicators of intestinal tight junction function, we wanted to investigate the response of the key intestinal site for absorption, the jejunum, to the effects of seaweed supplements in laying hens. The objective of this study was to determine the long-term effects in two commercial genetic strains of two seaweed supplements, *Chondrus crispus* and *Ascophyllum nodosum*, on OCC and ZO-1 protein expression in the jejunum of laying hens. 

## 2. Materials and Methods

### 2.1. Animal Trials and Sampling

Details of the animal trial and diet preparation were described previously [14,15]. Briefly, two strains of commercial laying hens, Lohmann LSL-Lite (White) and Lohmann Brown-Lite (Brown), which had been receiving identical diets, were assigned to continue either with the commercial mash diet (Control, Co) or a similar isocaloric, isonitrogenous diet supplemented with either 3% dried, ground red seaweed, *Chondrus crispus* (CC), or 0.5% dried, ground brown seaweed, *Ascophyllum nodosum* (AN, commercially available as Tasco^TM^). Diets were given in three phases over the lay period as recommended for commercial hens, and birds were housed in conventional battery cages with ad libitum food and water at 25 °C under a light program of 16 h of light/8 h of dark. 

Seaweed supplements were provided by Acadian Seaplants Ltd. (Halifax, NS, Canada). Levels of the supplements were chosen based on minimal levels associated with increased bird performance in our previous studies [13,14]. The 120 hens (24 cages of 5 each) were supplemented starting shortly after the onset of the peak laying period (31 weeks of age) to the normal end-of-lay time, 72 weeks of age, when samples were collected. The factorial complete randomized design included 2 genetic strains (White, Brown) × 3 diets (Co, CC, AN) × 4 replicates; the experimental unit was a cage of 5 hens. 

One bird from each cage was randomly selected at final sample time for euthanization. The middle section of jejunum was excised and quickly flushed with ice-cold phosphate-buffered saline (PBS). For immunohistochemistry and Western blotting, five segments approximately 1 cm in length were frozen in liquid nitrogen, and then stored at −80 °C. 

### 2.2. Cryosections and Immunohistochemistry

Cryostat cross-sections (5 μm in thickness) of jejunum were air-dried on Superfrost+ slides (Fisher Scientific Company, Ottawa, ON, Canada) and fixed with cold acetone for 10 min before air-drying and storage at −80 °C. For immunostaining, they were brought to room temperature, rehydrated in PBS for 10 min, treated with Bloxall^TM^ (Vector Laboratories, Burlingame, CA, USA) to quench endogenous enzyme activity, and washed again in PBS. Blocking of nonspecific antibody binding and detection of primary antibody binding were carried out as recommended for the Vector Elite ABC-HRP (Cat.#PK-7200) and ImmPACT AEC substrate kits (Vector Labs). Sections were incubated for 45 min with 1.25 mg/mL primary rabbit polyclonal antibody against occludin (Cat.# 711500, Fisher Scientific), 1.25 mg/mL rabbit polyclonal antibody ZO-1 (Cat.#617300, Fisher Scientific) or 1.25 mg/mL control rabbit IgG (Cat #026102, Fisher Scientific). 

At least three sections from each animal were immunostained on different experimental days. Independent, blind scoring of immunostaining was carried out on all sections to categorize major differences among treatments. Reactivity was scored from 1 (no staining) to 5 (extremely strong staining) of the intestinal epithelium, both of the luminal villus regions and deeper cryptal regions. Distribution was noted as apical, lateral or basally focused within the epithelium, and whether staining was more diffuse across the associated cells or membranes. It was noted that the jejunum samples varied in mucosal thickness and diameter, so cryosections were also scored (blind) for overall intestinal thickness. 

### 2.3. Western Blotting

Western blot analyses were performed to assess relative levels of occludin and ZO-1 in jejunal extracts in different animals by treatment. Jejunal samples were thawed and homogenized on ice with a cold T-Per Tissue Protein Extraction Reagent™ (Cat # 78510, Fisher Scientific Company, Ottawa, ON, Canada) supplemented with Halt™ protease inhibitor cocktail (Cat # 87786, Fisher Scientific). Protein concentration of samples was determined using the RC DC^TM^ Protein assay kit (BioRad Laboratories (Canada) Ltd., Mississauga, ON, Canada) with bovine serum albumin as a standard. Next, 20 μg of protein was solubilized in reducing sample buffer per the Western Blotting Application Solution Kit #12957 (Cell Signaling Technology, Danvers, MA, USA) and heated at 99.9 °C for 15 min. After denaturation, proteins were separated by SDS-PAGE in 4 to 20% (vol/vol) resolving gels under reducing conditions. Separated proteins were transferred to polyvinylidene difluoride membranes (BioRad) in Towbin buffer (25 mM Tris, 192 mM glycine, pH 8.4, 20% *v*/*v* methanol) at 100 V for 120 min. Nonspecific binding sites were blocked with a solution of non-fat dry milk (3%, *w*/*v*) containing 0.1% Tween 20 in tris-buffered saline (0.05-M Tris-HCl, 0.15-M NaCl, pH 7.6; TTBS) for one hour; then, the membrane was incubated with 12.5 mg/mL primary rabbit polyclonal antibody against occludin (Cat.# 711500, Fisher Scientific), 8.3 mg/mL rabbit polyclonal antibody ZO-1 (Cat.#617300, Fisher Scientific) or control rabbit IgG (Cat.#026102, Fisher Scientific) at the same concentration as the primary antibody. Following incubation at room temperature overnight and three washes in TTBS, antibody reactivity was demonstrated with the Immun-star goat anti-rabbit-AP Detection kit™ (BioRad) using chemiluminescence, and images were captured with a ChemiDoc™ XRS+ System (BioRad). Target protein bands were densitometrically scanned and adjusted for background protein level using Image Lab 2.0™ (Bio-Rad). Western blotting of samples from all 24 animals was repeated on three different dates.

### 2.4. Statistical Analysis

All statistical analyses were performed using Minitab 19 Statistical Software version 19.2020.1 [19]. The median thickness and immunohistochemical scores for each animal treatment combination were compared using nonparametric testing. The Mann–Whitney test was used to compare scores for staining intensity of villus epithelium to deeper cryptal epithelium. Mood’s median test was used to examine line and treatment differences in intestinal thickness and staining intensity. Chi-square analysis was used to examine treatment differences in mucosal thickness and staining distribution.

Occludin and ZO-1 Western blot band intensity values were subjected to square-root transformation to meet the assumption of normality; they then underwent the GLM procedure to test the effects of replicate (random effect) and the fixed effects of line and seaweed supplement. Tukey’s multiple comparison of means test was used to compare transformed treatment means. 

## 3. Results

**Figure 1:**
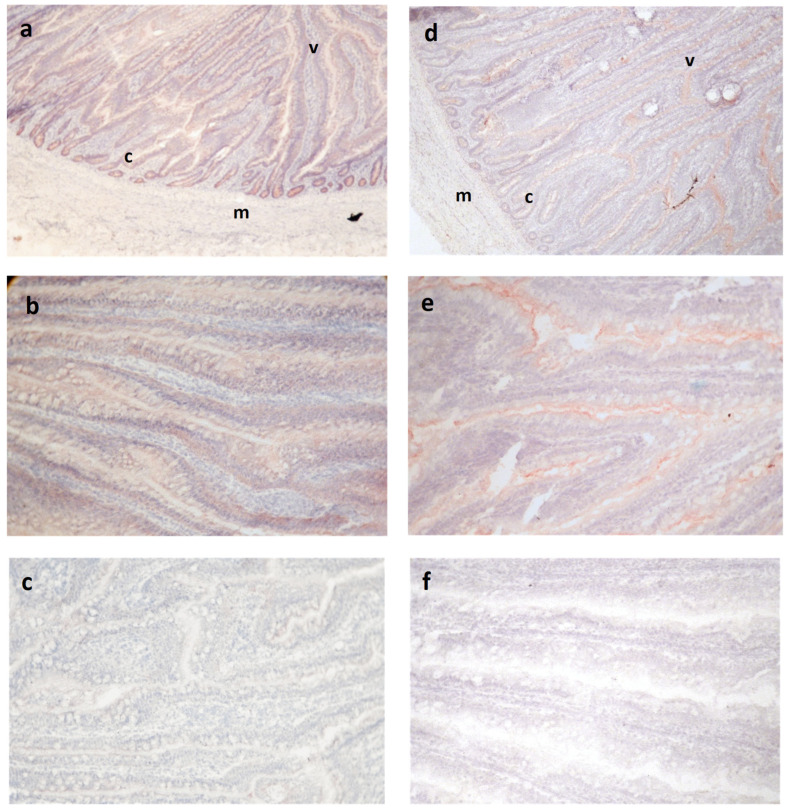
Tight junction regulatory protein immunoreactivity in hen jejunum. (**a**) Occludin (OCC) reactivity (red) is seen throughout the intestinal epithelium, appearing more intense in the deeper cryptal areas adjacent to the muscularis. (**b**) At higher magnification, OCC staining can be seen across the cell surface of the epithelial layer, often concentrated at the apicolateral cell surfaces. (**c**) OCC negative control. (**d**) Expression of zonula occludens-1 (ZO-1) protein in jejunal epithelium, appearing less pronounced in cryptal areas than the luminal villi. (**e**) Higher magnification shows ZO-1 reactivity is most pronounced at the apico-lateral cell surface. (**f**) ZO-1 negative control. v—villus; c—crypt; m—muscularis.

Occludin and ZO-1 antibodies reacted within intestinal epithelia but not stroma or muscular tissue, as expected for tight junction proteins, although the pattern of staining within the epithelium varied for the two antibodies (Figure 1). Occludin was observed across the epithelium in all animals, sometimes staining more strongly at the lateral cell surfaces of this layer but still maintaining a cytoplasmic distribution (Figure 1b). Within a cross-section of the jejunum, the cryptal epithelium appeared to stain more intensely than the luminal villus epithelium in most sections, but the Mann–Whitney comparison of their respective median scores did not show a significant difference (*p* = 0.47). 

Reactivity for ZO-1 was observed in all animals regardless of treatment or strain, although the pattern was different than for OCC (Figure 1c–e). Staining was concentrated on the apical aspect of the lateral epithelial cell membrane borders. Also, in contrast to OCC, reactivity for ZO-1 was less evident in the deeper crypt epithelium. 

There were no seaweed treatment effects on the intestinal thickness or staining intensity detected by immunohistochemistry for either OCC or ZO-1 (Table 1). In contrast, there may be strain effects on the staining intensity. The non-parametric Mood’s median test suggested that the villus epithelium of White hens expresses more OCC than in Brown hens (median intensity 3.5 vs. 2.5, respectively, *p* = 0.06). Conversely, White hens may express less ZO-1 in the deep cryptal epithelium (median intensity 1.5 vs. 2.5 in Brown hens, *p* = 0.06). 

**Figure 2:**
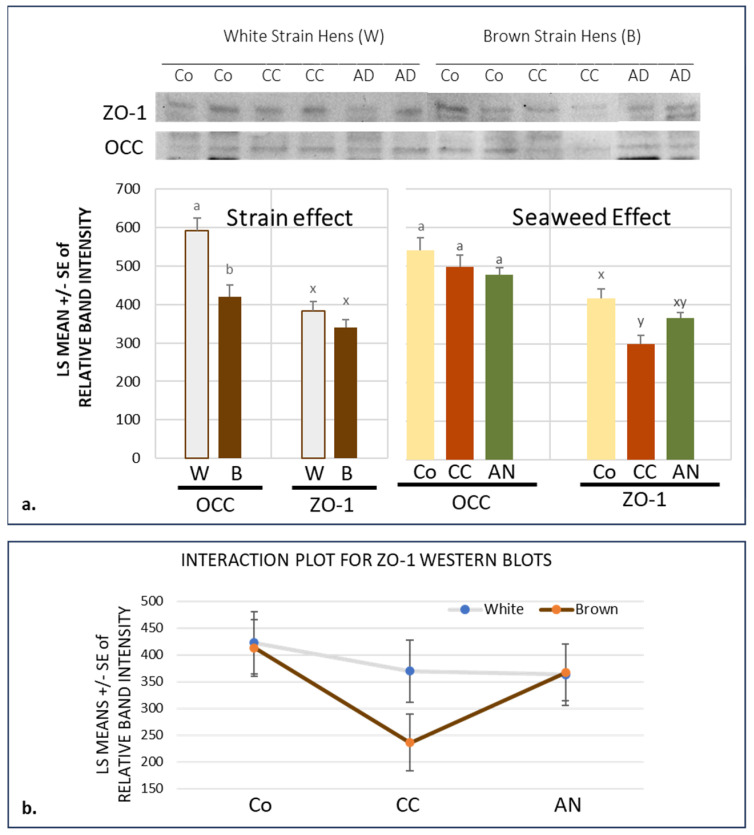
Western blot results of occludin (OCC) and zonula occludens-1 (ZO-1) protein expression in laying hen jejunum. Graphed results are least squares means ± standard error following Tukey’s means comparisons of square-root transformed relative band intensities. (**a**) Strain and seaweed supplement effects; within effect grouping, means with the same letter were not different (*p* < 0.05). (**b**) Strain x seaweed interaction effect on ZO-1 expression. Co—control diet; CC—*Chondrus crispus* supplement; AN—*Ascophyllum nodosum* supplement (See Supplementary Materials for original western blot information of Figure 2).

The chi-square analysis did not indicate any differences in the intestinal thickness score or staining pattern associated with bird strain or seaweed supplement (*p* > 0.05). Western blotting of the jejunal protein extracts showed that there were higher levels of occludin in White than Brown hen jejunal samples (*p* < 0.05; Figure 2). A decrease in the ZO-1 jejunal protein expression in Western blots was associated with *Chondrus crispus* supplementation in comparison to controls (*p* < 0.05), an effect that was more pronounced in Brown hens due to a supplement–strain interaction. *Ascophyllum nodosum* supplement effects were intermediate (*p* > 0.05).

## 4. Discussion

The pattern of OCC and ZO-1 reactivity in cryosections was consistent with previous studies, which showed ZO-1 being localized to the tight junctions close to the lumen of the cell, and OCC at cell junctions and more broadly in the intestinal epithelium [17,20]. Occludin is an integral membrane protein that was originally discovered in chicks as localizing to tight junctions, but it may redistribute to cytoplasmic vesicles and thus appear cytoplasmic [2,20,21]. The expression and distribution differ along the gastrointestinal tract and change with development [2,17]. The cytoplasmic distribution has been associated with a loss of barrier function and oxidative stress [3,20]. Others have shown reduced jejunal function in late lay hens [22]; these hens were 71 weeks of age in their late lay. It is possible that the diffuse distribution of OCC is associated with the hard metabolic work of laying over many months. This protein has several isoforms and functions differently depending on phosphorylation, including a role in mediating signals for apoptosis [5,6,21].

Our previous studies suggested that the more productive White layers are less sensitive than the Brown layers are to heat stress [13,14]. That OCC expression was higher in White than Brown layer strains would be consistent with an enhanced barrier function, and perhaps less susceptibility to age-related decline associated with the metabolic stress accumulating over a long laying period. Differences among breeds have been shown previously and may be part of the increased productivity response to genetic selection; commercial broilers showed better indicators of intestinal health and higher OCC levels than native Thai breeds in all three segments of the small intestine [17]. 

At the levels provided in this study, we anticipated neutral or beneficial effects of *Chondrus crispus* and *Ascophyllum nodosum* supplements. In broilers, AN has been shown to increase growth rates, improve the fatty acid profile and mitigate plasma indicators of inflammation [12]. In layers, CC improves egg quality, feed efficiency and indicators of intestinal function and decreases the microbial load in the digestive tract [12,13,14]. Our current results suggest that these positive effects are not related to increases in OCC and ZO-1 in the jejunum, although supplements that are considered beneficial to digestive health are often associated with higher OCC and ZO-1 expression in the jejunum and other parts of the digestive tract [9,23,24]. Reports of responses do vary; for example, supplemental tryptophan increased ZO-1 but not OCC in pig jejunum, and supplemental GOS increased both ZO-1 and OCC expression on day 8 but not on day 21 broilers [10,23]. 

While the overall ZO-1 expression was not significantly different between the two layer strains, seaweed supplement reduced the ZO-1 expression, particularly in the Brown hens. The observed CC seaweed-associated reduction in ZO-1 was surprising, since higher levels, not lower levels, were anticipated in response to a supplement that increased the villus height and surface area in previous studies [13]. Infection and other stressors that result in inflammation have frequently been shown to decrease ZO-1, which is thought to regulate the apical membrane structure and epithelial proliferation under stress. It has been proposed that pre- and probiotics can mitigate damage by mechanisms that increase or relocate OCC and ZO-1 [4,6,11,24]. However, as discussed above, there is complexity in responses and variability in responses along the digestive tract. Probiotics increased ZO-1 mRNA in broiler ileum but not in the jejunum [11]. Conversely, the probiotic GOS decreased jejunal ZO-1 in broilers, mitigating the heat-stress-associated increase that was observed in non-supplemented animals [10]. In the current trial, it is possible that the jejunal function in these late-lay-stage layers was more compromised in the Brown than White hens, and that the prebiotic seaweed was somehow mitigating the metabolic stress of extended lay through lowering the ZO-1 expression. It will be interesting to see if further studies confirm this observation.

## 5. Conclusions

Published studies on the impact of relatively short-term prebiotic supplements often show increases in OCC and/or ZO-1 as a positive marker of intestinal barrier function. In contrast, in this long-term study. the jejunum of late-stage laying hens showed a strain-dependent reduction in ZO-1 expression in response to long-term seaweed supplementation, as well as significant strain differences in the tight junction protein occludin’s expression levels. 

## Figures and Tables

**Table 1:** Median OCC and ZO-1 immunoreactivity scores for cryosections of laying hen jejunum.

	Seaweed Supplement ^1^				Genetic Strain		
	Control	CC	AN	Probability ^2^	White	Brown	Probability ^2^
No. of animals	8	8	8	—	12	12	—
OCC							
*Villus epithelium*	3	3.5	1.5	0.67	3.5	2.5	0.06
*Cryptal epithelium*	3	4	3	0.22	4	2.8	0.20
ZO-1							
*Villus epithelium*	2.5	3	2.5	0.32	2.5	2.5	0.86
*Cryptal epithelium*	2.5	2.5	2	0.76	1.5	2.5	0.06

^1^ CC—*Chondrus crispus*; AN—*Ascophyllum nodosum*. ^2^ Differences between medians subject to Mood’s median test; ns: *p* > 0.10.

## Data Availability

The data in the study are available in article or in previously published documents [12,14].

## Supplementary Materials

The following supporting information can be downloaded at: https://www.mdpi.com/article/10.3390/ani14050777/s1, Western blot original information of Figure 2.

## References

[1] Svihus B. (2014). Function of the digestive system. J. Appl. Poult. Res..

[2] Slifer Z.M., Blikslager A.T. (2020). The integral role of tight junction proteins in the repair of injured intestinal epithelium. Int. J. Mol. Sci..

[3] Awad W.A., Hess C., Hess M. (2017). Enteric pathogens and their toxin-induced disruption of the intestinal barrier through alteration of tight junctions in chickens. Toxins.

[4] Cani P.D., Possemiers S., Van de Wiele T., Guiot Y., Everard A., Rottier O., Geurts L., Naslain D., Neyrinck A., Lambert D.M. (2009). Changes in gut microbiota control inflammation in obese mice through a mechanism involving GLP-2-driven improvement of gut permeability. Gut.

[5] Cummins P.M. (2012). Occludin: One protein, many forms. Mol. Cell. Biol..

[6] Kuo W.-T., Odenwald M.A., Turner J.R., Zuo L. (2022). Tight junction proteins occludin and ZO-1 as regulators of epithelial proliferation and survival. Ann. N. Y. Acad. Sci..

[7] Obianwuna U.E., Qiu K., Wang J., Zhang H.J., Qi G.H., Huang L.L., Wu S.G. (2023). Effects of dietary *Clostridium butyricum* and fructooligosaccharides, alone or in combination, on performance, egg quality, amino acid digestibility, jejunal morphology, immune function, and antioxidant capacity of laying hens. Front. Microbiol..

[8] Guo Y., Zhao Z.H., Pan Z.Y., An L.L., Balasubramanian B., Liu W.C. (2020). New insights into the role of dietary marine-derived polysaccharides on productive performance, egg quality, antioxidant capacity, and jejunal morphology in late-phase laying hens. Poult. Sci..

[9] Tian S., Wang J., Yu H., Wang J., Zhu W. (2018). Effects of galacto-oligosaccarides on growth and gut function of newborn suckling piglets. J. Anim. Sci. Biotechol..

[10] Varasteh S., Braber S., Akbari P., Garssen J., Fink-Gremmels J. (2015). Differences in susceptibility to heat stress along the chicken intestine and the protective effects of galacto-oligosaccharides. PLoS ONE.

[11] Chang C.H., Teng P.Y., Lee T.T., Yu B. (2020). Effects of multi-strain probiotic supplemention on intestinal microbiota, tight junctions, and inflammation in young broiler chickens challenged with *Salmonella enterica* subsp. *enterica*. Asian-Australas. J. Anim. Sci..

[12] Makkar H.P.S., Tran G., Heuze V., Giger-Reverdin S., Lessire M., Lebas F., Anders P. (2016). Seaweeds for livestock diets: A review. Anim. Feed Sci. Technol..

[13] Kulshreshtha G., Rathgeber B., Stratton G., Thomas N., Evans F.J., Critchley A., Hafting J., Prithiviraj B. (2014). Feed supplementation with red seaweeds, *Chondrus crispus* and *Sarcodiotheca gaudichaudii*, affects performance, egg quality and gut microbiota of layer hens. Poult. Sci..

[14] Stupart C. (2019). Supplementation of Red Seaweed (*Chondrus crispus*) and Tasco^®^ (*Ascophyllum nodosum*) in Laying Hen Diets. Master’s Thesis.

[15] Borzouie S., Rathgeber B.M., Stupart C.M., MacIsaac J., MacLaren L.A. (2020). Effects of dietary inclusion of seaweed, heat stress and genetic strain on performance, plasma biochemical and hematological parameters in laying hens. Animals.

[16] Ekmay R.D., Chou K., Magnuson A., Lei X.G. (2015). Continual feeding of two types of microalgal biomass affected protein digestion and metabolism in laying hens. J. Anim. Sci..

[17] Theerawatanasirikul S., Koomkrong N., Kayan A., Boonkaewwan C. (2017). Intestinal barrier and mucosal immunity in broilers, Thai Betong, and native Thai Praduhangdum chickens. Turk. J. Vet. Anim. Sci..

[18] Wan Y., Ma R., Khalid A., Chai L., Qi R., Liu W., Li J., Li Y., Zhan K. (2021). Effect of the Pellet and Mash Feed Forms on the Productive Performance, Egg Quality, Nutrient Metabolism, and Intestinal Morphology of Two Laying Hen Breeds. Animals.

[19] (2022). Minitab. https://www.minitab.com.

[20] Kimura M., Sawada N., Kimura H., Isomura H., Hirata K., Mori M. (1996). Comparison between the distribution of 7H6 tight junction-associated antigen and occludin during the development of chick intestine. Cell Struct. Funct..

[21] Rao R. (2009). Occludin phosphorylation in regulation of epithelial tight junctions. Ann. N. Y. Acad. Sci..

[22] Gu Y.F., Chen Y.P., Jin R., Wang C., Wen C., Zhou Y.M. (2021). A comparison of intestinal integrity, digestive function, and egg quality in laying hens with different ages. Poult. Sci..

[23] Liu W., Mi S., Ruan Z., Li J., Shu X., Yao K., Jiang M., Deng Z. (2017). Dietary tryptophan enhanced the expression of tight junction protein ZO-1 in intestine. J. Food Sci..

[24] Wu Y., Yang F., Jiang W., Hu A., Xiong Z., Yang S., Cao P., Cao Z., Xiong Z., Cao H. (2022). Effects of compound probiotics on intestinal barrier function and caecum microbiota composition of broilers. Avian Pathol..

