# Supplementary material for: Changes in Tight Junction Protein Expression Levels but Not Distribution in Commercial White and Brown Laying Hens Supplemented with Chondrus crispus or Ascophyllum nodosum Seaweed
Source: Animals (Basel). 2024 Mar 1;14(5):777. doi: 10.3390/ani14050777 (PMC10931303; doi:10.3390/ani14050777)
Supplement: Supplementary file 1 [file animals-14-00777-s001.zip › animals-2837067-supplementary.pdf]

Figure 2 Western blot original info

Samples for Western Blots												Samples by treatment		
												ID#	Strain	Seaweed
ID Set-up Rep 1 & 2 Gels												1	W	Co
	**Lane 1**	**2**	**3**	**4**	**5**	**6**	**7**	**8**	**9**	**10**		2	W	CC
Gel 1	M	1	4	2	5	3	9	13	16	20		3	W	AN
Gel 2	M	7	10	8	11	12	25	20	23	21		4	W	Co
Gel 3	M	19	22	15	17	14	18	21	24	16		5	W	CC
ID Set-up Rep 3 Gels												7	W	Co
	**Lane 1**	**2**	**3**	**4**	**5**	**6**	**7**	**8**	**9**	**10**		8	W	CC
Gel 1	M	1	4	2	5	3	9	13	16	1		9	W	AN
Gel 2	M	7	10	8	11	12	25	20	23	7		10	W	Co
Gel 3	M	19	22	15	17	14	18	21	24	19		11	W	CC
												12	W	AN
												13	B	Co
												14	B	AN
												15	B	CC
												16	B	Co
												17	B	CC
												18	B	AN
												19	B	Co
												20	B	CC
												21	B	AN
												22	B	Co
												23	B	CC
												24	B	AN
												25	W	AN

All samples 1-24 w/ reblot of rep 1

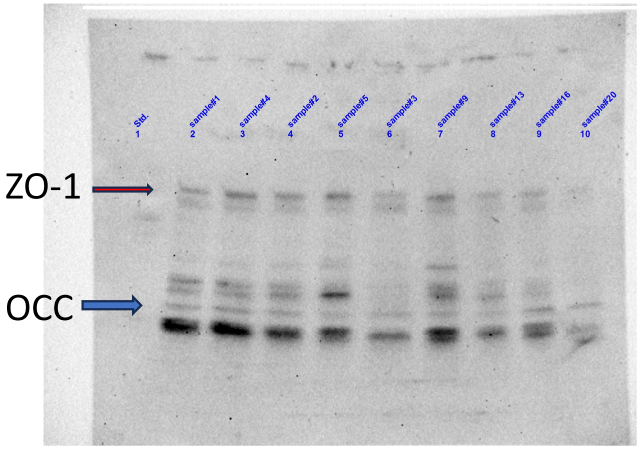

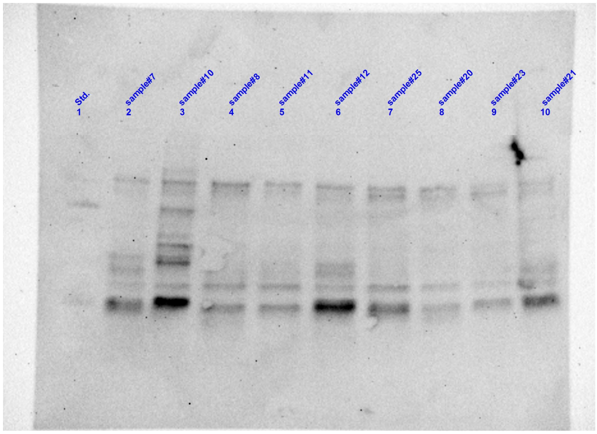

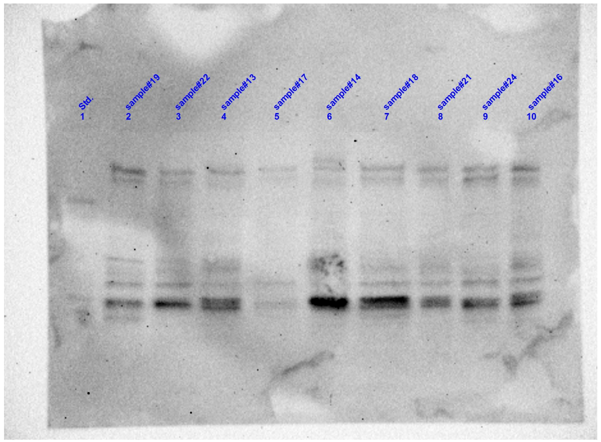

ZO-1- ~ 230 kDa
OCC - ~59 kDa

All samples 1-24 rep 2

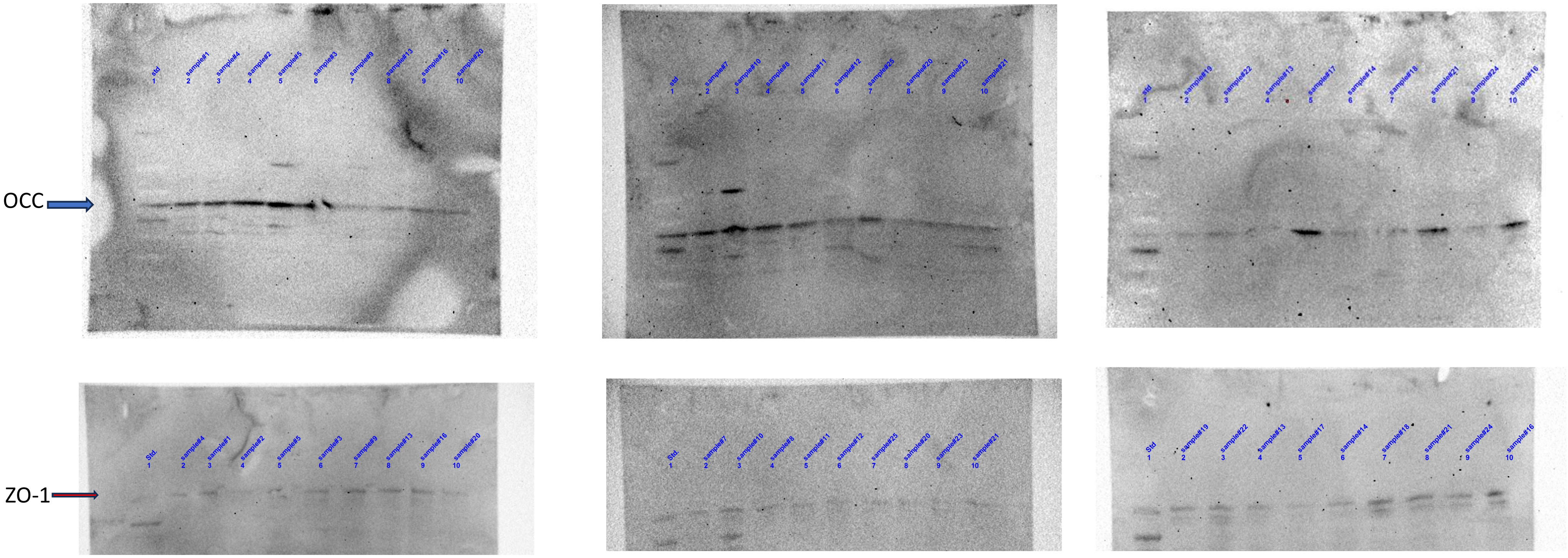

Trial blots

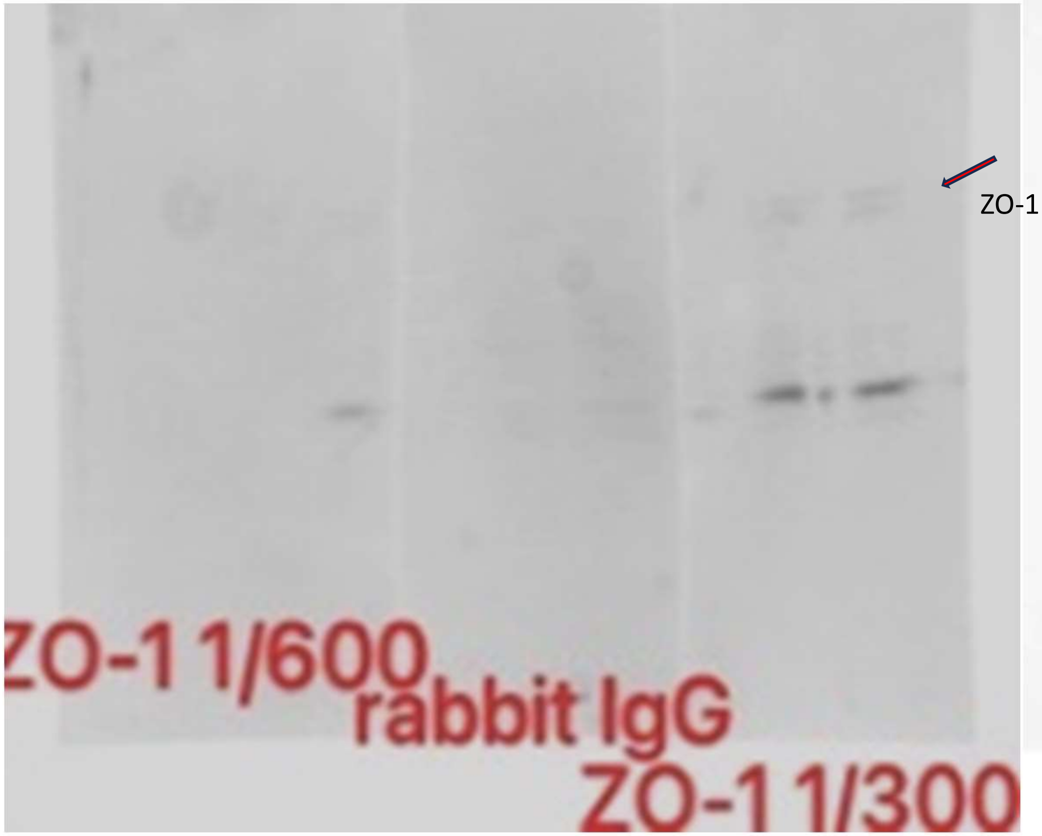

ZO-1
ZO-1 1/600
rabbit IgG
ZO-1 1/300

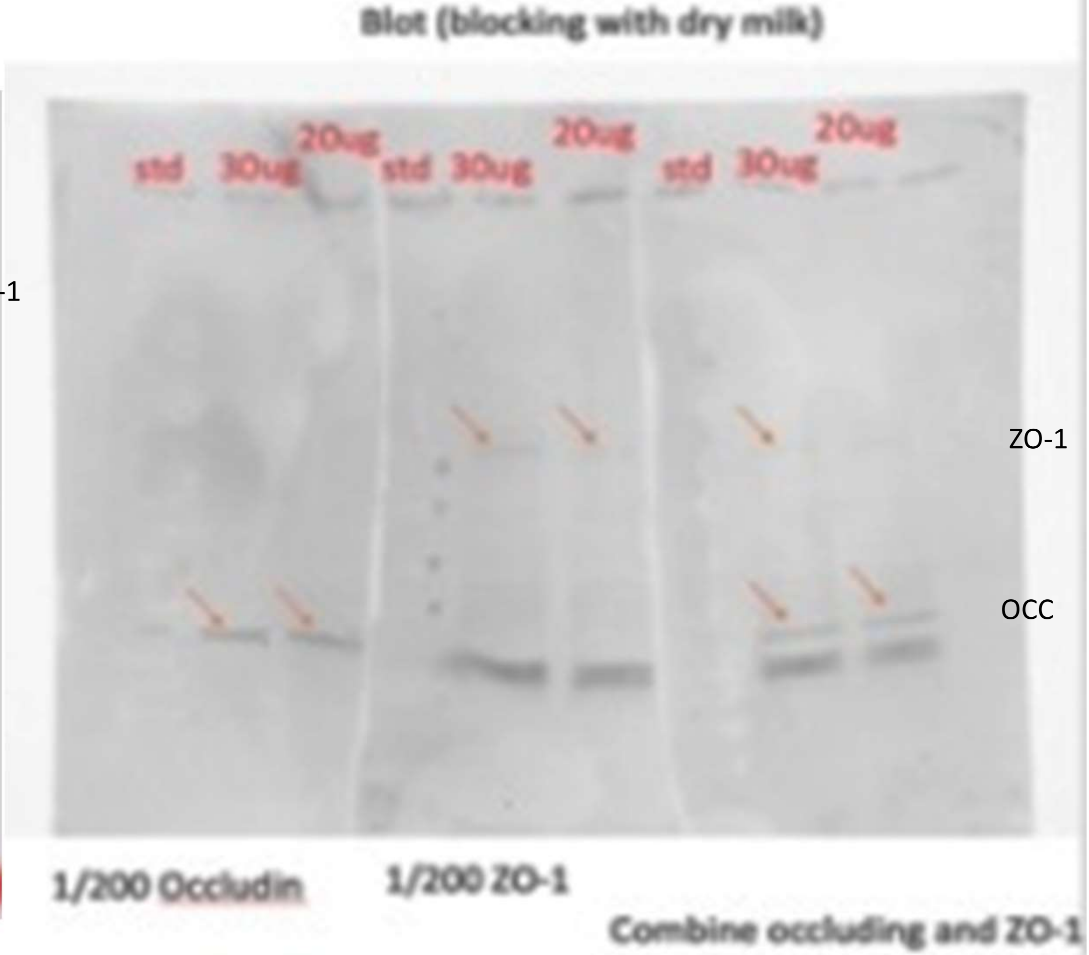

Blot (blocking with dry milk)
std
30ug
20ug
std
30ug
20ug
std
30ug
20ug
ZO-1
OCC
1/200 Occludin
1/200 ZO-1
Combine occluding and ZO-1
